# Mr. Barnard Holt’s Stricture Dilator

**Published:** 1852-09

**Authors:** 


*Mr. Barnard Holt's Stricture Dilator.*—Stricture, in all its forms, constitutes one of the most interesting varieties of lesion which the surgeon is called upon to treat; and although its origin and the minute anatomy of its early stages, have not yet, in some points, been fully explained and examined, yet a thorough and comprehensive knowledge of the treatment of it forms an indispensable branch of study for the practical surgeon.

Being confined to no one locality or class of patients, as well as being one of the most common forms of disease, it may call for aid in districts where little opportunity for studying surgery is afforded; while it may, and often does, severely tax the patience and skill of the great hospital surgeon.

As it is generally neglected by the patient till the last moment, and as no one of the various modes of treatment recommended seems available in all cases, the most energetic attempts have been made to arrive at sound conclusions, as well as to improve upon the instruments generally employed to dilate the contracted part of the canal. Among the latter, we may fairly notice those introduced to the Profession by Mr. T. Wakley.

Notwithstanding the advances made, great disunion of opinion prevails among the Profession as to the treatment of stricture, as was well illustrated at a meeting of the Medical and Chirurgical Society, held on the 13th of April of this year. A paper was read by Mr. Cock, containing the results of the treatment of stricture by puncture of the rectum. An elaborate and highly interesting discussion followed, in which nearly every speaker dissented from the views of those who had preceded him. Such discussions as these are of the greatest service to the Profession, and show how just a view the founders of them took respecting the steps then necessary for the advancement of science. They tell us where advances are made, and converts gained,—where opinions are still divided, and how objections might be raised or met; thus giving us, in one clear and condensed view, a summary of the opinions of some of the greatest surgeons and physicians of the day; opening up fresh sources of knowledge, and bringing to light facts and opinions which otherwise might die with their possessors. Hence the records of these societies and their debates may be consulted as storehouses of information, the value of which it is impossible to overrate, and the place of which no books or lectures can ever supply.

To enter into a full discussion of the various modes of treatment adopted in stricture would be here impracticable, and, if practicable, would be quite out of place. What occupied practised debaters some hours, could not be properly examined in the columns of a journal, nor appropriately appear in an article the functions of which are strictly limited to recording the discoveries of our hospital surgeons and physicians. We shall, therefore, proceed at once to the consideration of the subject in hand,—Mr. Holt's new instrument for the treatment of stricture, or, as the inventor has named it, the stricture dilator.

This extremely ingenious instrument is, Mr. Hall says, a modification of that contrived by Perreve. It consists of a tube like a catheter, split up to the extremity, where the two halves are welded together. Running through its whole length is a directing-rod; over this directing-rod can be passed a series of tubes of increasing calibre; thus affecting the slightest to the most extensive degree of dilatation. When the small closed tube is passed through the stricture, the sounds can be passed in succession, till the highest point is attained to which the surgeon wishes to go; and, as they are passed over the guiding-rod, and down the interior of the first instrument passed, it follows that all this dilatation may be attained without disturbance to the stricture. Here, then, may be said to be in some measure combined all the advantages which other instruments have ever offered. Steady, uniform dilatation, while the urethra is not irritated by renewed friction; the absence of any necessity for attempting to find again the difficult route; a continuance of the curative application, and thorough safety. The "stricture dilator" may therefore be fairly looked upon as a valuable addition to the means of treatment we possess. The following case will tend to show how satisfactorily it acts:—

J. G., a carpenter, aged 41, living in Lambeth, was admitted Jan., 1851, into Westminster Hospital. He gave the following account of his case. He had laboured under stricture twelve years. He knew of no cause for it, as the last gonorrhœa he ever remembers having suffered from had occurred eight years previously; but those who know how insidiously stricture springs up, and how obscure its origin is, will know how to appreciate these facts. However, having long suffered from it, he went at last to a surgeon, who attempted to introduce an instrument, but could not pass it more than two inches down. Considering it a very bad case, he recommended the patient to go into the hospital.

When admitted, the stream of urine was very narrow, not being thicker than a wire or knitting-needle, and no instrument could be passed into the bladder. An abscess formed in the perinæum, which was opened by Mr. Phillips. The patient was then transferred to the care of Mr. B. Holt, who commenced treating him with his stricture-dilator, Feb. 5, 1852.

Nothing larger than No. I could be passed at first. This, however, was got through every other day, but, owing to the appearance of some irritability and hæmorrhage, nearly a week's intermission in the use of instruments took place. The fourth time this dilatation was tried, the canal was found so much widened, the dilatation could be carried so far that a No. 6 was admitted. After this period, dilatation was steadily continued till towards the close of the month, when this report was drawn up. At this time, No. 9 could be passed with ease.

The man seemed very much pleased with the results of this treatment. The pain had been considerable at times, at others slighter; the abscess soon healed up, and the patient's health improved, especially as he could retain his urine in the night instead of being obliged to rise a dozen times to void it. These are satisfactory results in so short a time, and in a case where nearly the whole line of the canal was strictured.—*London Med. Timns.*

